# Single‐Particle Mid‐Infrared Photothermal Imaging Reveals Hidden Heterogeneity in Real‐World Micro‐ and Nanoplastics

**DOI:** 10.1002/advs.202524291

**Published:** 2026-04-27

**Authors:** Xinyu Deng, Yongqing Zhang, Xiaobin Tang, Hyeon Jeong Lee, Delong Zhang

**Affiliations:** ^1^ Zhejiang Key Laboratory of Micro‐nano Quantum Chips and Quantum Control School of Physics Zhejiang University Hangzhou China; ^2^ College of Biomedical Engineering & Instrument Science Key Laboratory for Biomedical Engineering of Ministry of Education Zhejiang University Hangzhou China

**Keywords:** chemical imaging, microplastics, microstructural heterogeneity, nanoplastics, single‐particle analysis

## Abstract

Micro‐ and nanoplastics are increasingly detected in human tissues, yet quantitative methods capable of resolving their physicochemical heterogeneity and linking such diversity to functional consequences remain limited. Bottled water, a major ingestion route, provides a controlled matrix for investigating real‐world nanoplastic contamination. Here, we employ mid‐infrared photothermal (MIP) microscopy to achieve single‐particle chemical and morphological characterization, revealing substantial heterogeneity at both microstructural and particle scales. Multidimensional spectral analysis of polyethylene terephthalate (PET), the dominant polymer, uncovers pronounced spectral narrowing, indicating enhanced intra‐particle uniformity with physicochemical features distinct from laboratory standards. Population‐level spectral measurements further show continuous variations in PET crystallinity, demonstrating the nonuniform nature of environmental particles. Morphological profiling resolves discrete size and shape distributions across polymer types, enabling differentiation of contamination sources and highlighting regulatory blind spots in manufacturing. Collectively, these results show that particles of similar size and composition can occupy divergent physicochemical states. Such property‐resolved insights are essential for accurate exposure assessment and for guiding predictive models, mitigation strategies, and future standards for micro‐ and nanoplastic monitoring.

## Graphical abstract

Mid‐infrared photothermal imaging enables multidimensional profiling of micro‐ and nanoplastics in bottled water. A total of 9.9 × 10^4^ particles L^−1^ is detected, with 64% in the nanoscale regime. Spectral evolution, including peak narrowing and band shifts, reveals local chain reorganization in polyethylene terephthalate (PET), highlighting intrinsic structural heterogeneity at the single‐particle level.

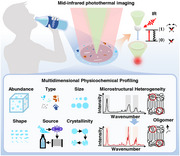

## 1 Introduction

Micro‐nanoplastics (MNPs) pollution has emerged as a pressing global environmental and health challenge, with plastic particles now unequivocally detected in multiple human tissues [1, 2, 3, 4, 5, 6]. Although causal links between MNPs and disease remain under investigation, accumulating evidence demonstrates that polymer type, shape, and size fundamentally determine cellular uptake, toxicity, and interactions with biological systems [7, 8]. Comprehensive physicochemical characterization is therefore critical for exposure assessment, particularly for nanoplastics (NPs, < 1 µm) that can traverse biological barriers via multiple endocytic pathways [9]. However, NPs remain poorly understood due to limitations in both representative models and analytical methods.

Current studies often rely on pristine, monodisperse polymer spheres that poorly capture the physic‐chemical complexity of real‐world nanoplastics, which have typically undergone extensive mechanical abrasion, chemical weathering, and other environmental transformations [10, 11]. Analytical methods further constrain understanding. Bulk compositional methods such as pyrolysis‐GC‐MS obscure population‐level heterogeneity by averaging over many particles [12]. Size‐based analyses, such as nanoparticle tracking analysis (NTA), cannot distinguish whether particles of identical dimensions share chemical composition or represent distinct degradation stages [13]. Conventional imaging approaches sacrifice either chemical specificity (electron microscopy) or spatial resolution (FTIR, ∼10 µm) [14, 15]. These limitations preclude the mapping of single‐particle chemical variations that underpin biological behavior.

Mid‐infrared photothermal (MIP) microscopy, a high‐resolution vibrational imaging technique, provides a powerful means to address these limitations. By detecting IR‐induced refractive index changes with a visible laser, MIP achieves submicron spatial resolution while inheriting the chemical richness of established FTIR spectral libraries [16, 17, 18]. Although MIP has been applied to diverse polymeric matrices [19, 20, 21, 22, 23, 24, 25, 26, 27, 28], systematic studies of nanoplastics using this approach are still lacking.

To bridge both the model and methodological gaps, we employ bottled water as a controlled yet environmentally relevant system. Bottled water constitutes a major ingestion pathway, with global consumption exceeding 350 billion liters in 2023 (45 billion liters in China) [29, 30, 31], and recent analyses reporting up to 2.4 × 10^5^ particles L^−1^, 90% of which are NPs [32]. Its low background contamination and defined composition make it an ideal platform for single‐particle chemical imaging.

**FIGURE 1:**
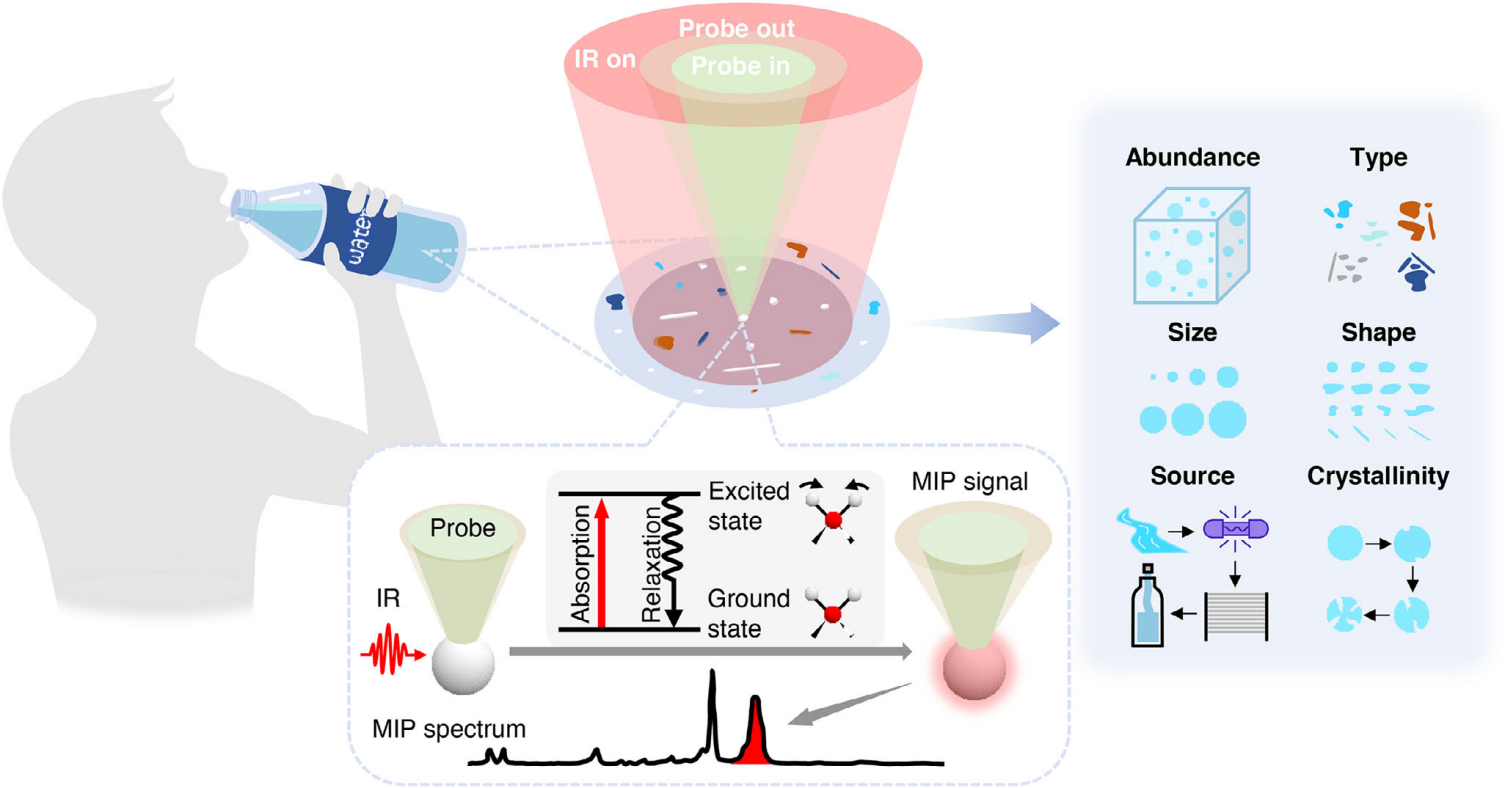
Conceptual framework for chemical mapping of nanoplastics in bottled water using MIP microscopy. The approach uncovers molecular and morphological heterogeneity essential for exposure and risk assessment.

Here, we use MIP microscopy to characterize nanoplastics from three major Chinese bottled water brands (Figure 1). We quantified the levels of MNP contamination in bottled water, with NPs comprising over 60% of particles. The morphological characteristics of particle populations within each polymer type exhibited high heterogeneity, enabling source attribution. Population‐level spectroscopic analysis reveals that polyethylene terephthalate (PET) dominates contamination (∼90% by count) and displays notable spectral heterogeneity against PET standards as well as variations in physicochemical states among the particles. These findings establish that exposure assessments based solely on particle counts or size distributions underestimate true heterogeneity and provide valuable reference data for human exposure assessment and toxicological modeling.

## 2 Results

### 2.1 Wavelength‐Selective Imaging Resolves Nanoscale Contamination Across Macroscopic Filters

**FIGURE 2:**
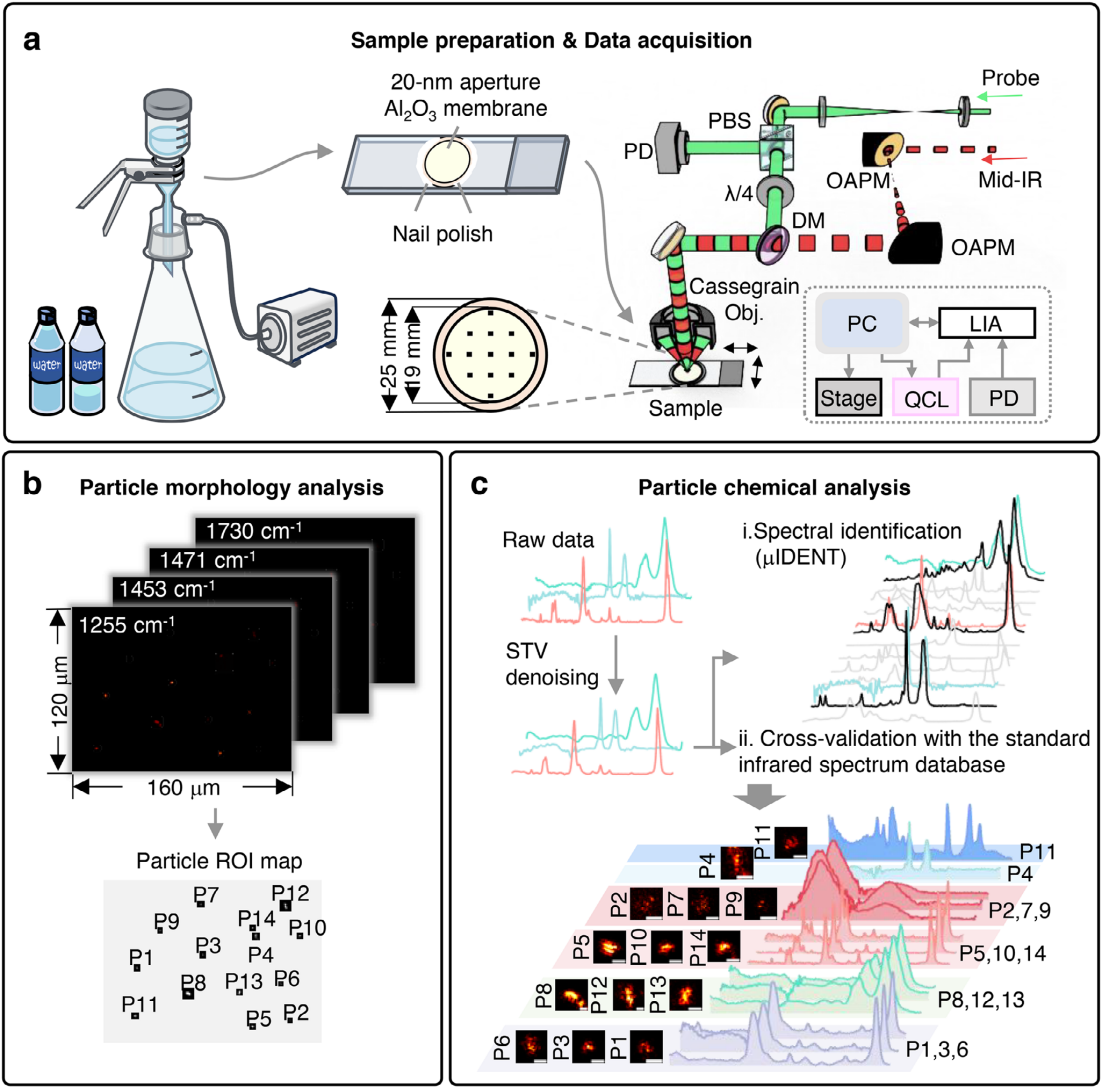
Workflow for sample processing, data acquisition, and analysis of MNPs in bottled water. (a) Sample preparation and MIP microscopy setup. Plastic particles from bottled water were collected by vacuum filtration through a 20‐nm Al_2_O_3_ membrane. Representative fields of views (160 × 120 µm^2^ each) were acquired. Dashed box: electronic wiring. PBS: polarizing beam splitter, PD: photodetector, DM: dichroic mirror, OAPM: off‐axis parabolic mirror, λ/4: quarter‐wave plate, QCL: quantum cascade laser, LIA: lock‐in amplifier. (b) Images at C─O─C stretching, CH_3_ bending, CH_2_ bending, and C═O stretching frequencies were acquired and then summed to generate particle ROI maps. (c) Spectra extracted from masked regions were normalized and denoised using the spectral total variation (STV) algorithm, then matched against an MIP spectral database or standard infrared library for polymer identification. µIDENT: microplastics identification algorithm. Scale bars, 2 µm.

Characterizing bottled water contamination requires spatially‐resolved spectroscopy across centimeter‐scale filters containing sparsely distributed sub‐micron particles, presenting a challenge that would require impractical acquisition times using conventional hyperspectral imaging. We implemented a two‐stage workflow (Figure 2a,b): wavelength‐selective scanning at diagnostic polymer frequencies (C─O─C stretching vibration at 1255 cm^−1^, CH_2_/CH_3_ bending vibration at 1453/1471 cm^−1^, C═O stretching vibration at 1730 cm^−1^; Figure S1) rapidly locates particles across representative 160 × 120 µm^2^ fields, followed by full mid‐IR spectral acquisition (933–1799 cm^−1^) at identified particle locations. Summed wavelength‐selective images processed by intensity thresholding simultaneously extract particle positions and morphological parameters (size, shape), while spectra matched against polymer reference libraries including our collections (Figure S2) and a public database [33] (Figure 2c), with key peak positions listed in Table S1. This integrated approach delivers comprehensive single‐particle characterization, including chemical composition, morphology, and structural signatures. Validation experiments using procedural blanks (Milli‐Q water), clean membrane controls, and PMMA microsphere standards (150–500 nm) confirmed detection sensitivity to 150 nm with a signal‐to‐noise ratio > 3.7 and minimal contamination (Figure S3).

### 2.2 Consumer‐Relevant NP Population for Heterogeneity Characterization

**FIGURE 3:**
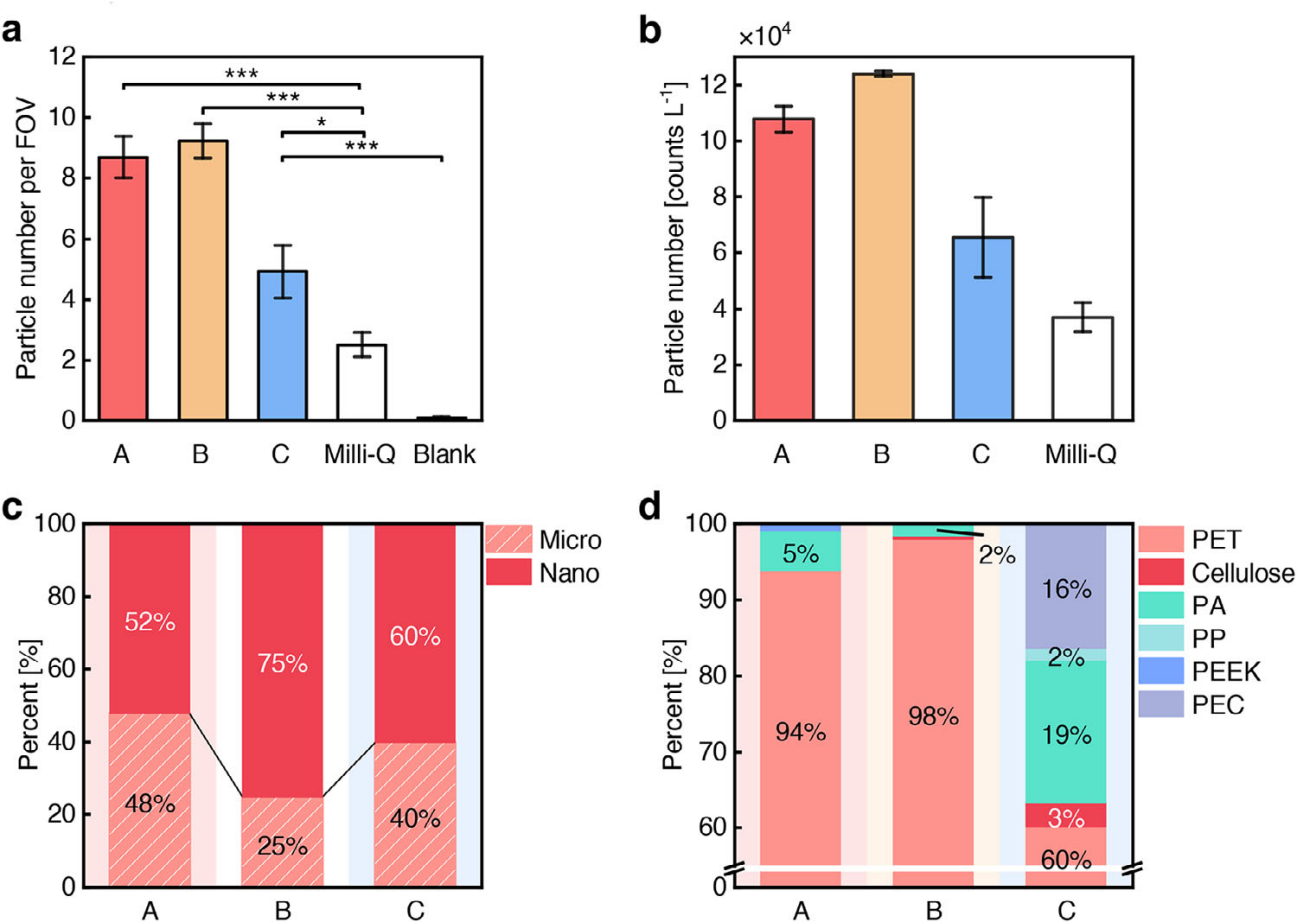
Quantification of MNP contamination in bottled water. (a) Mean number of particles per field of view (error bars: mean ± SEM; significant determined by two‐tailed *t*‐test: n = 26, * 0.01 < *P* < 0.05, *** *P* < 0.001). (b) Estimated particle counts per liter of bottled water (n = 2, error bars, mean ± SEM). (c) Size distribution showing the proportion of MPs and NPs across brands. (d) Polymer composition of detected particles in each brand.

To test whether consumer‐relevant NP exposures comprise heterogeneous populations, we characterized bottled water as a representative high‐volume exposure pathway. Given the broad size distributions and irregular morphologies of environmentally derived particles, we adopt the minimum Feret diameter as the primary size descriptor, defined as the minimum distance between two parallel lines enclosing the object. Particles with a minimum Feret diameter < 1 µm were defined as NPs and those ≥1 µm as MPs. Plastic particle contamination reached 9.9 ± 1.2 × 10^4^ particles L^−1^ across three Chinese brands (Figure 3a,b), with NPs (64%, 6.3 ± 0.8 × 10^4^ L^−1^) dominating over MPs (36%, 3.6 ± 0.6 × 10^4^ L^−1^; Figure 3c). This particle burden, detected across representative sampling (13 fields per filter), provides a statistically adequate population for chemical heterogeneity analysis.

The measured MP concentration (3.6 × 10^4^ particles L^−1^) aligns with previous studies reporting 10^3^–10^4^ MPs L^−1^ in bottled water [34, 35, 36, 37, 38, 39, 40, 41]. Extending the analysis to smaller size regimes, concurrent NP quantification reveals substantially higher total particle burdens than MP‐only assessments. Comparing with recent U.S. bottled water data obtained using stimulated Raman scattering (SRS) microscopy shows agreement in the order of magnitude of total contamination levels (2.4 × 10^5^ total particles L^−1^, ∼90% NPs) [32]. Such agreement, despite substantial differences in sampling and analytical methodologies, supports the quantitative consistency of vibrational imaging. Notably, both studies demonstrate that NPs comprise > 60% of particle burden, suggesting that MP‐focused monitoring systematically underestimates exposure by one to two orders of magnitude.

Four polymer types accounted for 99.3% of detected particles (Figure 3d): polyethylene terephthalate (PET, 88.2%), polyamide (PA, 6.7%), polyether carbonate (PEC, 3.5%), and cellulose (∼1%). PET (the bottle material) dominated contamination and exhibited structural heterogeneity spectral signatures (see below). Notably, PEC, an eco‐friendly polymer synthesized from CO_2_ and used in battery applications [42, 43], was identified in bottled water for the first time (detected exclusively in Brand C), suggesting potential watershed‐level environmental contamination.

Polypropylene (PP), the primary cap material, was detected at remarkably low abundance (2 particles across 78 fields analyzed), despite extensive water‐cap contact during storage. This minimal presence contrasts with glass‐bottled beverages, where cap‐derived paint particles dominate contamination [44], suggesting that PP release mechanisms differ fundamentally from paint abrasion and that bottle material degradation represents the primary NP source in PET‐bottled water.

### 2.3 Population‐Level Heterogeneity: Polymer‐Specific Morphological Distributions

**FIGURE 4:**
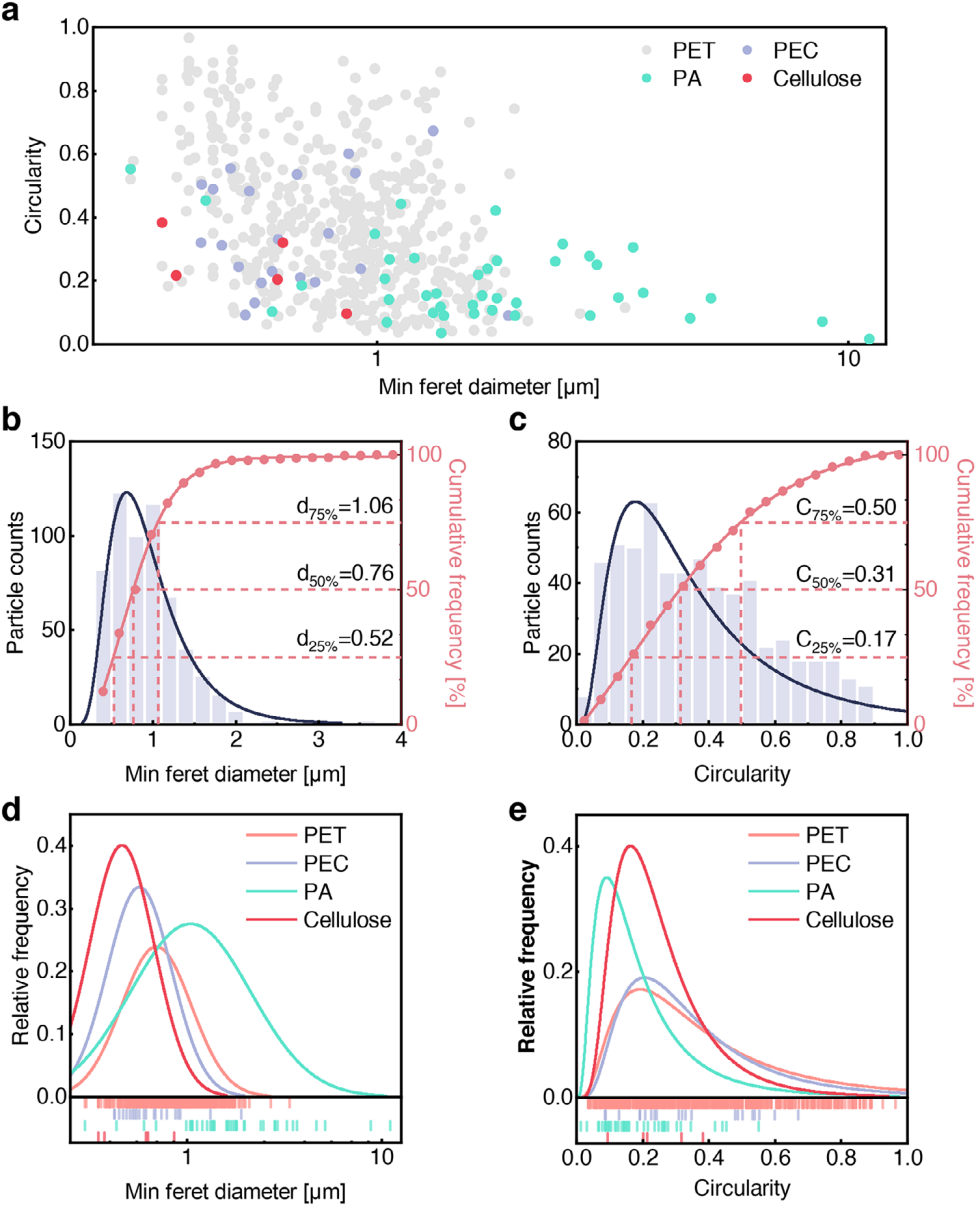
Morphological heterogeneity in consumer‐relevant NP populations. (a) Two‐dimensional scatter plot of circularity versus minimum Feret diameter for identified MNPs. (b, c) Population‐level size and shape distributions (log‐normal fit) and cumulative frequency (Boltzmann fit) reveal pronounced polydispersity and deviation from spherical geometries. (d, e) Polymer‐specific distributions (log‐normal fit) enable source attribution as a secondary application.

Single‐particle chemical‐morphological analysis revealed pronounced population‐level heterogeneity. Since traditional morphological categories (fiber, fragment) are ambiguous at the nanoscale, we quantified shape using circularity (4π × Area/Perimeter^2^, where 1.0 = perfect sphere; Figure S4). Scatter analysis of circularity versus minimum Feret diameter showed distinct polymer clustering (Figure 4a): PET spans 0.04‐0.97 circularity across 300–3300 nm; PEC clusters at small sizes (< 1900 nm) and low circularity (< 0.5); PA occupies large dimensions (0.3–11 µm) with uniformly irregular shapes; cellulose forms small (< 900 nm), highly elongated particles. These polymer‐specific distributions demonstrate that particle morphology reflects release mechanism and subsequent transformations rather than generic size‐dependent fragmentation.

This morphological heterogeneity has direct consequences for cellular interactions that uniform‐particle models cannot capture. Across all particles, the minimum Feret diameter averaged 990 ± 740 nm (mean ± SD) while circularity averaged 0.37 ± 0.22 (mean ± SD; Figure 4b,c), indicating predominantly irregular/elongated morphologies. Deviation from spherical geometry affects multiple exposure‐relevant properties simultaneously: (1) increased specific surface area relative to equivalent‐volume spheres enhances adsorption of co‐contaminants (e.g., heavy metals and persistent organic pollutants), altering exposure composition [45, 46]; (2) shape‐dependent uptake is well established, with spheres preferentially entering via clathrin‐mediated endocytosis while elongated or highly anisotropic particles more frequently utilize macropinocytosis or phagocytosis, so shape‐heterogeneous particle populations can activate multiple internalization pathways [47, 48, 49]; (3) elongated particles are prone to induce frustrated phagocytosis and exhibit prolonged biopersistence compared with spherical particles [50, 51]. Consequently, the morphological heterogeneity documented here indicates that real‐world NP exposures engage cellular machinery differently than monodisperse spherical particles used in laboratory studies.

Beyond establishing exposure heterogeneity, polymer‐specific morphological distributions enabled contamination source attribution (Figure 4d,e). PET particles (900 ± 380 nm, circularity 0.39 ± 0.22) exhibited dimensions and structural heterogeneity signatures (Section 2.4) consistent with packaging material breakdown. Water treatment infrastructure contributed 7.6% of particles through two distinct sources: cellulose particles (570 ± 210 nm, 0.24 ± 0.11 circularity) matching nanofiber dimensions from cellulose‐based prefilters, and PA particles (2.26 ± 2.09 µm, 0.19 ± 0.12 circularity) corresponding to reverse osmosis membrane fragments [32]. PEC (710 ± 350 nm, 0.35 ± 0.18 circularity), detected exclusively in Brand C, likely represents watershed‐level environmental contamination from industrial sources [42, 43]. This source attribution capability demonstrates practical utility: identifying contamination pathways enables targeted intervention strategies.

### 2.4 Distinct Vibrational Signatures Reveal Structural Heterogeneity in Released PET Particles

**FIGURE 5:**
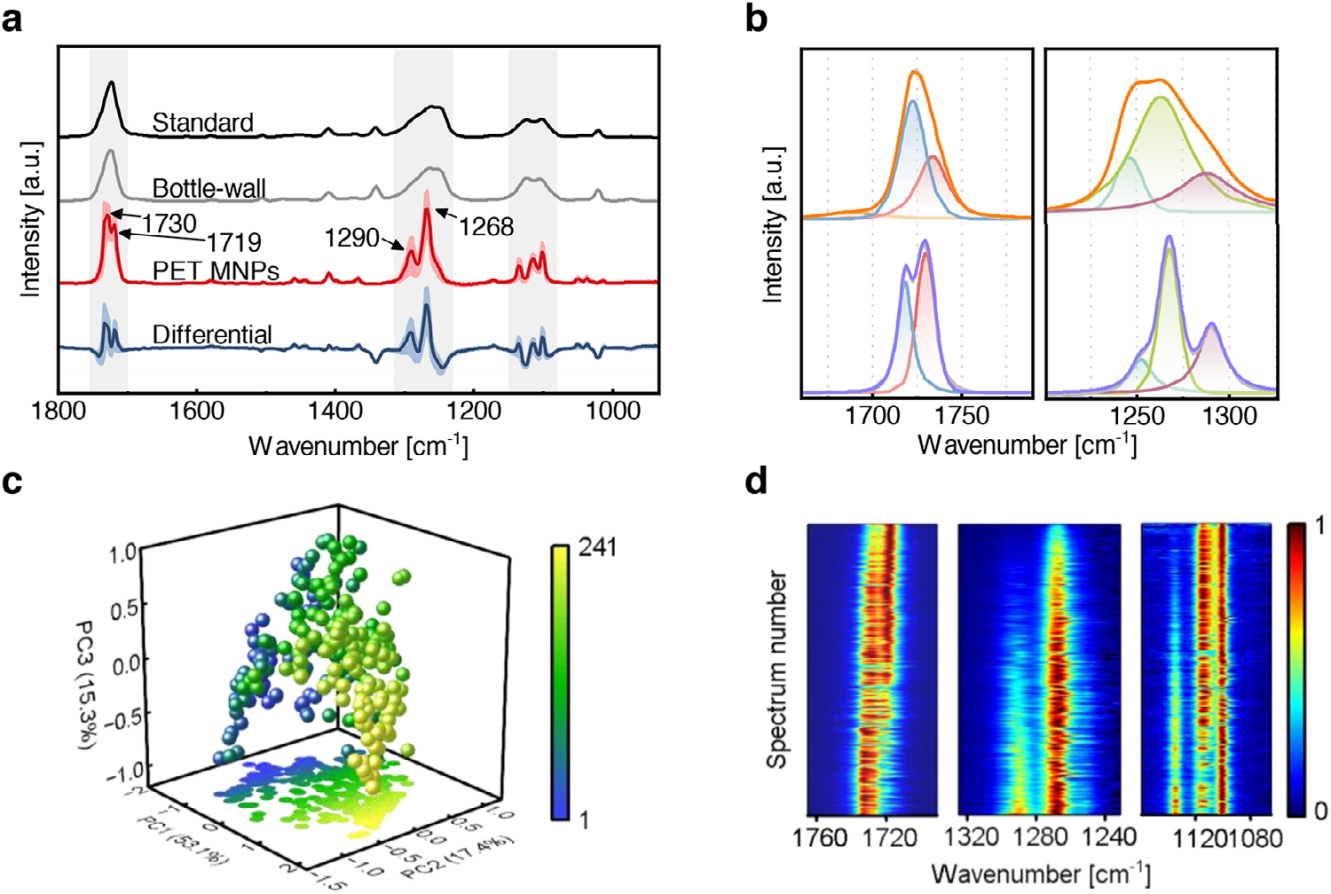
Spectroscopic signatures and statistical analysis revealing structural heterogeneity in PET particles in bottled water. (a) MIP spectra of PET standards, bottle‐wall PET, and PET MNPs, together with their corresponding differential spectra. All spectra are normalized to the 1410 cm^−1^ C─H aromatic ring band. Solid lines represent mean spectra, and shaded areas denote the interquartile range (IQR). (b) Peak‐fitting results of the carbonyl and ester C─O─C bands for bottle‐wall PET (top) and PET MNPs (bottom), using a Gaussian‐Lorentzian cross function (R^2^ > 0.99). Gray curves show the raw spectra, and orange and purple curves represent the cumulative fitted profiles. (c) PCA score plot of 241 PET MNP spectra. (d) Spectral intensity maps of the 1719–1730 cm^−1^ carbonyl band, the 1250–1290 cm^−1^ ester C─O─C region, and the 1100–1136 cm^−1^ conformational region, ordered along PC1. The carbonyl band and 1250–1290 cm^−1^ region is min‐max normalized together, whereas the 1100–1136 cm^−1^ region is normalized independently.

Comparison of 241 high‐SNR spectra from PET particles in bottled water with commercial PET standards and bottle‐wall PET shows that the released particles possess distinct and highly reproducible spectral features. After normalization to the C─H aromatic ring vibration at 1410 cm^−1^ band [52, 53], the commercial standard and bottle‐wall PET exhibit nearly identical spectral envelopes, whereas the particles display pronounced band splitting at the ∼1720 cm^−1^ carbonyl band, the 1250–1290 cm^−1^ ester C─O─C region, and the 1100–1136 cm^−1^ conformational region. These differences remain evident in the corresponding difference spectra of PET particle spectra and bottle wall spectra (Figure 5a).

Narrowing of the vibrational bands provides a consistent explanation for the clearly resolved peak splitting. Peak‐fitting analysis shows that the carbonyl band separates into two sub‐bands at 1719 and 1730 cm^−1^ (Figure 5b), matching the well‐established dual assignments used to distinguish crystalline and amorphous C═O environments in PET [54]. In the 1250–1290 cm^−1^ ester C─O─C region, three sub‐peaks are resolved, which have exhibited independently evolving sub‐band intensities during PET crystallization in previous research [55], supporting the interpretation that the deconvolved peaks represent distinct local conformations or crystallinity‐related vibrational modes. All these sub‐bands exhibit markedly reduced full width at half maximum (FWHM) in the particles compared with the bottle‐wall PET. Such peak narrowing typically reflects a convergence of local vibrational environments and therefore provides an indicator of increased local molecular homogeneity. Moreover, the sub‐band centers display systematic shifts of 2.5–6 cm^−1^ between the two sample types, which are well above the fitting uncertainty (Table S2), indicating measurable changes in chain packing or segmental structure. Notably, the ∼1690 cm^−1^ C═O vibration, a characteristic vibration of PET monomers or oligomers observed in the bottle‐wall PET (Figure S5) [56], becomes strongly attenuated or absent in the particles. Prior studies have demonstrated that PET oligomers and low‐molecular‐weight species can migrate or be released during hydrolysis [11, 57]. Therefore, this phenomenon is consistent with the leaching or depletion of low‐molecular‐weight species into the aqueous phase. Finally, while the 1100–1120 cm^−1^ region is generally considered a double‐like feature, we resolve a three‐peak structure for the first time. The bottle‐wall PET cannot produce a stable three‐peak fit under the same criteria, indicating that additional, previously unresolved vibrational modes become detectable in the particles. Collectively, these observations suggest that during the transport and storage of bottled water, PET particles may undergo both the migration or loss of oligomers or other low‐molecular‐weight species and hydrothermal‐induced local chain rearrangement or ordering.

The significant peak narrowing characteristic enhances sensitivity to crystallinity‐ and conformation‐dependent features. The relatively large standard deviations observed in the 1100–1136, 1250–1290, and 1719–1730 cm^−1^ regions reflect spectral heterogeneity among particles. Principal component analysis (PCA) of these bands shows a continuous distribution along the first principal component, rather than discrete clustering (Figure 5c), corresponding to systematic changes in the intensity ratio of the fitted carbonyl sub‐peaks and the characteristic ester bands. Sorting the spectra along this component reveals a gradual shift of the carbonyl band from 1730 to 1719 cm^−1^, accompanied by a systematic decrease in the bands at 1268 and 1290 cm^−1^ (Figure 5d). These spectral variations are consistent with differences in molecular ordering and crystallinity among particles. The continuous distribution suggests nonuniform structural states that may arise during the release process of PET, reflecting heterogeneous degradation or reorganization histories. In addition, the relative weakening of the 1136 cm^−1^ band and strengthening of the 1115 cm^−1^ band suggest conformation‐dependent contributions, where the 1136 cm^−1^ feature may be associated with trans or amorphous structures, and the 1115 cm^−1^ band with cis or more ordered (crystalline) configurations.

Overall, the PET particles released into bottled water are not simple fragments of pristine plastic. They exhibit systematic band narrowing, altered local vibrational environments, and pronounced structural heterogeneity. These characteristics demonstrate that real‐world PET particles develop distinct molecular fingerprints, which may influence their stability, environmental transport, and biological interactions. Such complexity is often overlooked when studies rely solely on commercial or laboratory standards. These findings highlight the importance of incorporating environmentally derived particles into future exposure and toxicological assessments, since their structural and chemical states differ fundamentally from those of commercial materials.

To ensure that the observed structural features are not influenced by photothermal excitation during measurement, we performed physics‐based thermal modeling and experimental validation. The modeling shows that, for 1‐µm PET particles in air, the maximum temperature rise is ∼8 K, and decays to ambient conditions within ∼10 µs (Figure S7), remaining well below the threshold required to induce macromolecular chain reorganization. Experimentally, we collected 22 consecutive spectra at the same location on a single PET particle over a total duration of 220 s, substantially exceeding the exposure time used during routine experiments. The spectra show no evidence of band splitting or peak narrowing, with a spectral similarity exceeding 99% (Figure S8). These results indicate that the observed structural features are intrinsic, rather than artifacts arising from photothermal excitation.

## 3 Discussion

Our integrated chemical and morphological analysis establishes that consumer‐relevant nanoplastic exposures are heterogeneous at two scales. At the molecular level, particles exhibit systematic features, including narrowed and resolved sub‐peaks in fingerprint‐region vibrational bands, which are absent in bulk materials and pristine references. As established in prior studies and discussed above [54, 55], such spectral features are associated with variations in molecular ordering and crystallinity. These features therefore, reflect variations in local molecular environments within individual particles. At the particle level, particle population spans a continuous spectrum of spectroscopic states. Based on established relationships between vibrational features and molecular ordering, as well as variations in band intensity, this distribution is consistent with variations in crystallinity. This heterogeneity challenges the common practice of treating size‐composition categories as equivalent exposure units in toxicity studies and risk models.

The observed heterogeneity in particle properties has direct implications for toxicological assessment. First, PET with different crystallinity levels exhibits distinct surface properties and mechanical properties, affecting the adhesion strength and internalization process of the granular cells [58, 59]. Second, laboratory toxicity studies overwhelmingly employ pristine, compositionally uniform particles or artificially aged variants. Dose‐response relationships derived from such uniform materials cannot predict biological responses to heterogeneous environmental populations where particle crystallinity can vary substantially. Third, particles released from bulk materials may exhibit heterogeneous microstructures, consistent with processes such as hydrothermal‐induced local chain rearrangement or ordering during particle release, neither of which is represented in current testing paradigms. Extrapolating from uniform laboratory particles to consumer‐relevant exposures requires assumptions about property‐toxicity relationships that remain empirically unvalidated.

Notably, the abundance of MNPs measured here is consistent in order of magnitude with recent results obtained using SRS microscopy [32]. Despite substantial differences in sampling strategies and analytical workflows, this level of agreement is stronger than typically expected and supports the quantitative robustness of vibrational imaging for MNP characterization. MIP microscopy provides sub‐micron spatial resolution with full fingerprint‐region coverage, enabling simultaneous characterization of particle abundance, chemical identity, and morphology. It further resolves intra‐particle spectral variations associated with molecular ordering, linking population‐level distributions to underlying structural heterogeneity at the single‐particle level.

Alongside vibrational imaging approaches, single‐particle electrochemical sensing technologies, including nanopore and nanoelectrochemical methods, enable high‐sensitivity characterization of individual nanoscopic entities across a broad size range, extending to sub‐10‐nm particles and even single molecules [60, 61]. These approaches extract physical information, such as size, charge, and interfacial properties, from ionic current or electrochemical signals and are well suited for resolving dynamic heterogeneity at the single‐entity level [62]. Mid‐infrared photothermal imaging, in turn, directly probes molecular bonds and enables chemically specific characterization over extended spatial regions. This capability enables high‐throughput analysis of sparsely distributed particles over large fields of view, which is essential for studying environmentally derived MNPs and biological samples. Together, these approaches provide complementary perspectives on the physical and chemical properties of heterogeneous particle populations.

Beyond mechanistic and toxicological implications, comprehensive single‐particle characterization also enables practical source attribution. Polymer‐specific morphological distributions enabled source attribution: PET particles (88% of total, 900 ± 380 nm) exhibited thermal degradation signatures consistent with packaging breakdown; water treatment infrastructure contributed 7.6% through cellulose prefilter (570 ± 210 nm) and polyamide RO membrane (2.26 ± 2.09 µm) fragmentation. These results reveal regulatory blind spots that elevated temperatures during bottled water transport and storage may accelerate particle release and fragmentation, whereas filtration systems are validated only for contaminant removal, not for particle generation. Additionally, PEC detection exclusively in Brand C suggests watershed‐level environmental contamination, distinguishing source‐water‐specific inputs from packaging‐derived particles.

The heterogeneity documented here establishes essential reference data while revealing critical knowledge gaps. Future toxicity studies should employ environmentally relevant particle populations spanning observed property distributions rather than uniform surrogates, enabling construction of predictive models linking particle characteristics to biological outcomes. Regulatory frameworks for nanoscale particulates must account for heterogeneity in both exposure assessment (property distributions, not point values) and hazard characterization (population‐based rather than single‐particle toxicity data). The 9.9 × 10^4^ particles L^−1^ contamination level we measured, with NPs comprising 64% of the burden, provides baseline data for exposure models. However, the pronounced chemical and morphological heterogeneity within this population demands that risk assessments move beyond treating size‐composition categories as equivalent units.

The present work still has limitations. The 20‐nm filtration membrane sets a lower bound on detectable particle size; thus, the reported particle abundance and heterogeneity reflect only the retained fraction. Extending detection to sub‐100 nm particles remains an important goal, which we envision could be addressed in future work by far‐field super‐resolution vibrational imaging techniques [17, 63]. Another challenge is associated with the sparse distribution of MNPs. While filtration enriches particles from dilute samples, they remain sparsely distributed on the membrane, necessitating large‐area screening. This places stringent demands on imaging throughput, which can be addressed by laser‐scanning mid‐infrared photothermal microscopy. [64]

## Funding

National Key Research and Development Program of China (2024YFA1408900), Russian Science Foundation (26‐79‐31011), National Science Foundation of China (W2532057, 82372011), Zhejiang Provincial Natural Science Foundation of China (LZ25H180001), and Fundamental Research Funds for the Central Universities (226‐2025‐00034).

## Conflicts of Interest

The authors declare no conflicts of interest.

## Supporting information




**Supporting File**: advs75475‐sup‐0001‐SuppMat.docx.

## Data Availability

The data that support the findings of this study are available from the corresponding author upon reasonable request.

## References

[1] A. J. Nihart , M. A. Garcia , E. El Hayek , et al., “Bioaccumulation of Microplastics in Decedent Human Brains,” Nature Medicine 31, no. 4 (2025): 1114–1119, 10.1038/s41591-024-03453-1. PMC12003191 39901044

[2] Y. Yang , E. Xie , Z. Du , et al., “Detection of Various Microplastics in Patients Undergoing Cardiac Surgery,” Environmental Science & Technology 57, no. 30 (2023): 10911–10918, 10.1021/acs.est.2c07179. 37440474

[3] T. Horvatits , M. Tamminga , B. Liu , et al., “Microplastics Detected in Cirrhotic Liver Tissue,” eBioMedicine 82 (2022): 104147, 10.1016/j.ebiom.2022.104147. 35835713 PMC9386716

[4] R. Marfella , F. Prattichizzo , C. Sardu , et al., “Microplastics and Nanoplastics in Atheromas and Cardiovascular Events,” New England Journal of Medicine 390, no. 10 (2024): 900–910, 10.1056/NEJMoa2309822. 38446676 PMC11009876

[5] Q. Yang , Y. Peng , X. Wu , et al., “Microplastics in Human Skeletal Tissues: Presence, Distribution and Health Implications,” Environment International 196 (2025): 109316, 10.1016/j.envint.2025.109316. 39946929

[6] A. Ragusa , A. Svelato , C. Santacroce , et al., “Plasticenta: First Evidence of Microplastics in Human Placenta,” Environment International 146 (2021): 106274, 10.1016/j.envint.2020.106274. 33395930

[7] S. Lambert , C. Scherer , and M. Wagner , “Ecotoxicity Testing of Microplastics: Considering the Heterogeneity of Physicochemical Properties,” Integrated Environmental Assessment and Management 13, no. 3 (2017): 470–475, 10.1002/ieam.1901. 28440923

[8] J. Zhao , R. Lan , Z. Wang , et al., “Microplastic Fragmentation by Rotifers in Aquatic Ecosystems Contributes to Global Nanoplastic Pollution,” Nature Nanotechnology 19, no. 3 (2024): 406–414, 10.1038/s41565-023-01534-9. 37945989

[9] R. Lehner , C. Weder , A. Petri‐Fink , and B. Rothen‐Rutishauser , “Emergence of Nanoplastic in the Environment and Possible Impact on Human Health,” Environmental Science & Technology 53, no. 4 (2019): 1748–1765, 10.1021/acs.est.8b05512. 30629421

[10] P. Pfohl , M. Wagner , L. Meyer , et al., “Environmental Degradation of Microplastics: How to Measure Fragmentation Rates to Secondary Micro‐ and Nanoplastic Fragments and Dissociation Into Dissolved Organics,” Environmental Science & Technology 56, no. 16 (2022): 11323–11334, 10.1021/acs.est.2c01228. 35902073 PMC9387529

[11] N. F. Mendez , V. Sharma , M. Valsecchi , et al., “Mechanism of Quiescent Nanoplastic Formation From Semicrystalline Polymers,” Nature Communications 16, no. 1 (2025): 3051, 10.1038/s41467-025-58233-3. PMC11953330 40155643

[12] Z. Huang , E. Wu , D. Shi , et al., “Six Microplastics Analysis in Bottled Water, Purified Tap Water and Branded Table Salt by Double‐Shot Pyrolysis–Gas Chromatography/Mass Spectrometry,” Chromatographia 87, no. 10 (2024): 675–683, 10.1007/s10337-024-04359-3.

[13] L. M. Hernandez , E. G. Xu , H. C. E. Larsson , R. Tahara , V. B. Maisuria , and N. Tufenkji , “Plastic Teabags Release Billions of Microparticles and Nanoparticles Into Tea,” Environmental Science & Technology 53, no. 21 (2019): 12300–12310, 10.1021/acs.est.9b02540. 31552738

[14] J.‐L. Xu , K. V. Thomas , Z. Luo , and A. A. Gowen , “FTIR and Raman Imaging for Microplastics Analysis: State of the Art, Challenges and Prospects,” TrAC Trends in Analytical Chemistry 119 (2019): 115629, 10.1016/j.trac.2019.115629.

[15] A. Winkler , F. Fumagalli , C. Cella , D. Gilliland , P. Tremolada , and A. Valsesia , “Detection and Formation Mechanisms of Secondary Nanoplastic Released From Drinking Water Bottles,” Water Research 222 (2022): 118848, 10.1016/j.watres.2022.118848. 35901554

[16] D. Zhang , C. Li , C. Zhang , M. N. Slipchenko , G. Eakins , and J.‐X. Cheng , “Depth‐Resolved Mid‐Infrared Photothermal Imaging of Living Cells and Organisms With Submicrometer Spatial Resolution,” Science Advances 2, no. 9 (2016): 1600521, 10.1126/sciadv.1600521. PMC5040478 27704043

[17] P. Fu , W. Cao , T. Chen , et al., “Super‐Resolution Imaging of Non‐Fluorescent Molecules by Photothermal Relaxation Localization Microscopy,” Nature Photonics 17, no. 4 (2023): 330–337, 10.1038/s41566-022-01143-3.

[18] J.‐X. Cheng , Y. Yuan , H. Ni , et al., “Advanced Vibrational Microscopes for Life Science,” Nature Methods 22, no. 5 (2025): 912–927, 10.1038/s41592-025-02655-w. 40360912

[19] S. L. Belontz , J. Brahney , C. E. Caplan , E. Dillon , T. Yan , and G. Dominguez , “Combining Submicron Spectroscopy Techniques (AFM‐IR and O‐PTIR) to Detect and Quantify Microplastics and Nanoplastics in Snow From a Utah Ski Resort,” Environmental Science & Technology 59, no. 26 (2025): 13362–13373, 10.1021/acs.est.4c12170. 40569162 PMC12243121

[20] R. L. Parham , A. M. Ayala , L. Meagher , et al., “Identifying Microplastics in Laboratory and Atmospheric Aerosol Mixtures via Optical Photothermal Infrared and Raman Microspectroscopy,” Analytical Chemistry 97, no. 33 (2025): 18136–18143, 10.1021/acs.analchem.5c02968. 40810689

[21] Y. Su , X. Hu , H. Tang , et al., “Steam Disinfection Releases Micro(Nano)Plastics From Silicone‐Rubber Baby Teats as Examined by Optical Photothermal Infrared Microspectroscopy,” Nature Nanotechnology 17, no. 1 (2022): 76–85, 10.1038/s41565-021-00998-x. 34764453

[22] A. Tarafdar , J. Xie , A. Gowen , A. C. O'Higgins , and J.‐L. Xu , “Advanced Optical Photothermal Infrared Spectroscopy for Comprehensive Characterization of Microplastics From Intravenous Fluid Delivery Systems,” Science of The Total Environment 929 (2024): 172648, 10.1016/j.scitotenv.2024.172648. 38649036

[23] Y. Su , C. Yang , H. Liao , et al., “Microplastic Contamination of Human Sperm Before in Vitro Fertilization Warrants Attention for Early Life Exposure Risks,” Environment & Health 3 (2025): 1208–1219. 41127840 10.1021/envhealth.5c00098 PMC12538326

[24] X. Lin , A. A. Gowen , S. Chen , and J.‐L. Xu , “Baking Releases Microplastics From Polyethylene Terephthalate Bakeware as Detected by Optical Photothermal Infrared and Quantum Cascade Laser Infrared,” Science of The Total Environment 924 (2024): 171408, 10.1016/j.scitotenv.2024.171408. 38432360

[25] F. Yan , X. Wang , H. Sun , et al., “Development of a Binary Digestion System for Extraction Microplastics in Fish and Detection Method by Optical Photothermal Infrared,” Frontiers in Marine Science 9 (2022): 845062, 10.3389/fmars.2022.845062.

[26] L. Costello , A. Zetterström , P. Gardner , et al., “Microplastics Accumulate in all Major Organs of the Mediterranean Loggerhead Sea Turtle (Caretta Caretta),” Marine Environmental Research 208 (2025): 107100, 10.1016/j.marenvres.2025.107100. 40203720

[27] K. Duswald , V. Pichler , V. Kopatz , et al., “Detection of Unlabeled Polystyrene Micro‐ and Nanoplastics in Mammalian Tissue by Optical Photothermal Infrared Spectroscopy,” Analytical Chemistry 97, no. 31 (2025): 16714–16722, 10.1021/acs.analchem.4c05400. 40749980 PMC12356189

[28] J.‐R. Macairan , A. Saherwala , F. Li , F. Monteil‐Rivera , and N. Tufenkji , “Label‐Free Identification and Imaging of Microplastic and Nanoplastic Biouptake Using Optical Photothermal Infrared Microspectroscopy,” Environmental Science & Technology 59, no. 30 (2025): 15612–15622, 10.1021/acs.est.4c14367. 40696889 PMC12330824

[29] Statista Market Share of the Leading Bottled Water Brands in China in 2023 (Statista, 2024), https://www.statista.com/statistics/880080/china‐leading‐bottled‐water‐brand‐market‐share/.

[30] Credence Research China Bottled Water Market Size, Share and Forecast 2032 (Credence Research, 2025), https://www.credenceresearch.com/report/china‐bottled‐water‐market.

[31] K. K. Maharjan , “Microplastic Pollution in Bottled Water: A Systematic Review,” International Journal of Environmental Science and Technology 22, no. 2 (2025): 1283–1296, 10.1007/s13762-024-05807-1.

[32] N. Qian , X. Gao , X. Lang , et al., “Rapid Single‐Particle Chemical Imaging of Nanoplastics by SRS Microscopy,” Proceedings of the National Academy of Sciences 121, no. 3 (2024): 2300582121, 10.1073/pnas.2300582121. PMC10801917 38190543

[33] “Shanghai Institute of Organic Chemistry of CAS,” 2025, https://organchem.csdb.cn.

[34] Y.‐T. Tse , S. M.‐N. Chan , and E. T.‐P. Sze , “Quantitative Assessment of Full Size Microplastics in Bottled and Tap Water Samples in Hong Kong,” International Journal of Environmental Research and Public Health 19, no. 20 (2022): 13432, 10.3390/ijerph192013432. 36294013 PMC9602710

[35] H. LI , L. ZHU , M. MA , H. WU , L. AN , and Z. YANG , “Occurrence of Microplastics in Commercially Sold Bottled Water,” Science of The Total Environment 867 (2023): 161553, 10.1016/j.scitotenv.2023.161553. 36640894

[36] S. A. Mason , V. G. Welch , and J. Neratko , “Synthetic Polymer Contamination in Bottled Water,” Frontiers in Chemistry 6 (2018): 407, 10.3389/fchem.2018.00407. 30255015 PMC6141690

[37] D. Kankanige and S. Babel , “Smaller‐Sized Micro‐Plastics (MPs) Contamination in Single‐Use PET‐Bottled Water in Thailand,” Science of The Total Environment 717 (2020): 137232, 10.1016/j.scitotenv.2020.137232. 32062244

[38] F. Nacaratte , P. Cuevas , M. Becerra‐Herrera , and C. A. Manzano , “Early Screening of Suspected Microplastics in Bottled Water in the Santiago Metropolitan Region of Chile,” Environmental Pollution 334 (2023): 122118, 10.1016/j.envpol.2023.122118. 37414125

[39] P. Zhou , K. Zhang , T. Zhang , C. Cen , Y. Zheng , and Y. Shuai , “Release Characteristics of Small‐Sized Microplastics in Bottled Drinks Using Flow Cytometry Sorting and Nile Red Staining,” Water 16, no. 13 (2024): 1898, 10.3390/w16131898.

[40] C. Vitali , R. J. B. Peters , H.‐G. Janssen , et al., “Quantitative Image Analysis of Microplastics in Bottled Water Using Artificial Intelligence,” Talanta 266 (2024): 124965, 10.1016/j.talanta.2023.124965. 37487270

[41] B. E. Oßmann , G. Sarau , H. Holtmannspötter , M. Pischetsrieder , S. H. Christiansen , and W. Dicke , “Small‐Sized Microplastics and Pigmented Particles in Bottled Mineral Water,” Water Research 141 (2018): 307–316, 10.1016/j.watres.2018.05.027. 29803096

[42] Y. Tominaga , “Ion‐Conductive Polymer Electrolytes Based on Poly(Ethylene Carbonate) and Its Derivatives,” Polymer Journal 49, no. 3 (2017): 291–299, 10.1038/pj.2016.115.

[43] Y. Sasanuma and Y. Takahashi , “Structure–Property Relationships of Poly(ethylene carbonate) and Poly(propylene carbonate),” ACS Omega 2, no. 8 (2017): 4808–4819, 10.1021/acsomega.7b00964. 31457761 PMC6641952

[44] I. Chaïb , P. Doyen , P. Merveillie , A. Dehaut , and G. Duflos , “Microplastic Contaminations in a Set of Beverages Sold in France,” Journal of Food Composition and Analysis 144 (2025): 107719, 10.1016/j.jfca.2025.107719.

[45] N. Rafa , B. Ahmed , F. Zohora , et al., “Microplastics as Carriers of Toxic Pollutants: Source, Transport, and Toxicological Effects,” Environmental Pollution 343 (2024): 123190, 10.1016/j.envpol.2023.123190. 38142809

[46] L.‐C. Wang , J. C.‐T. Lin , J.‐A. Ye , et al., “Enrichment of Persistent Organic Pollutants in Microplastics From Coastal Waters,” Environmental Science & Technology 58, no. 50 (2024): 22391–22404, 10.1021/acs.est.4c10835. 39629940 PMC11656714

[47] C. Kinnear , T. L. Moore , L. Rodriguez‐Lorenzo , B. Rothen‐Rutishauser , and A. Petri‐Fink , “Form Follows Function: Nanoparticle Shape and Its Implications for Nanomedicine,” Chemical Reviews 117, no. 17 (2017): 11476–11521, 10.1021/acs.chemrev.7b00194. 28862437

[48] J. J. Rennick , A. P. R. Johnston , and R. G. Parton , “Key Principles and Methods for Studying the Endocytosis of Biological and Nanoparticle Therapeutics,” Nature Nanotechnology 16, no. 3 (2021): 266–276, 10.1038/s41565-021-00858-8. 33712737

[49] M. Sousa de Almeida , E. Susnik , B. Drasler , P. Taladriz‐Blanco , A. Petri‐Fink , and B. Rothen‐Rutishauser , “Understanding Nanoparticle Endocytosis to Improve Targeting Strategies in Nanomedicine,” Chemical Society Reviews 50, no. 9 (2021): 5397–5434, 10.1039/D0CS01127D. 33666625 PMC8111542

[50] L. A. Visani de Luna , T. Loret , Y. He , et al., “Pulmonary Toxicity of Boron Nitride Nanomaterials Is Aspect Ratio Dependent,” ACS Nano 17, no. 24 (2023): 24919–24935, 10.1021/acsnano.3c06599. 38051272 PMC10753895

[51] A. Vogel , J. Tentschert , R. Pieters , et al., “Towards a Risk Assessment Framework for Micro‐ and Nanoplastic Particles for Human Health,” Particle and Fibre Toxicology 21, no. 1 (2024): 48, 10.1186/s12989-024-00602-9. 39614364 PMC11606215

[52] K. Kawahara , H. Matsuno , and K. Tanaka , “Hygrothermal Degradation Behavior and Structural Evolution of Electrospun Poly(Ethylene Terephthalate) Fiber Mats,” Langmuir 41, no. 34 (2025): 23238–23244, 10.1021/acs.langmuir.5c03382. 40844126 PMC12409890

[53] R. A. Wilkes , N. Zhou , A. L. Carroll , et al., “Mechanisms of Polyethylene Terephthalate Pellet Fragmentation Into Nanoplastics and Assimilable Carbons by Wastewater *Comamonas* ,” Environmental Science & Technology 58, no. 43 (2024): 19338–19352, 10.1021/acs.est.4c06645. 39360733 PMC11526368

[54] Z. Chen , J. N. Hay , and M. J. Jenkins , “The Kinetics of Crystallization of Poly(Ethylene Terephthalate) Measured by FTIR Spectroscopy,” European Polymer Journal 49, no. 6 (2013): 1722–1730, 10.1016/j.eurpolymj.2013.03.020.

[55] Z. Chen , J. N. Hay , and M. J. Jenkins , “FTIR Spectroscopic Analysis of Poly(Ethylene Terephthalate) on Crystallization,” European Polymer Journal 48, no. 9 (2012): 1586–1610, 10.1016/j.eurpolymj.2012.06.006.

[56] S. Ubeda , M. Aznar , and C. Nerín , “Determination of Oligomers in Virgin and Recycled Polyethylene Terephthalate (PET) Samples by UPLC‐MS‐QTOF,” Analytical and Bioanalytical Chemistry 410, no. 9 (2018): 2377–2384, 10.1007/s00216-018-0902-4. 29428989

[57] T. Yang , Y. Xu , G. Liu , and B. Nowack , “Oligomers Are a Major Fraction of the Submicrometre Particles Released During Washing of Polyester Textiles,” Nature Water 2, no. 2 (2024): 151–160, 10.1038/s44221-023-00191-5.

[58] S. Wieland , A. F. R. M. Ramsperger , W. Gross , et al., “Nominally Identical Microplastic Models Differ Greatly in Their Particle‐Cell Interactions,” Nature Communications 15, no. 1 (2024): 922, 10.1038/s41467-024-45281-4. PMC10830523 38297000

[59] M. L. Di Lorenzo , “Crystallization of Poly(Ethylene Terephthalate): A Review,” Polymers 16, no. 14 (2024): 1975, 10.3390/polym16141975. 39065291 PMC11280767

[60] K.‐L. Chen , Y.‐L. Ying , A. G. Ewing , and Y.‐T. Long , “Nanopipette Electrochemistry,” Chemical Reviews 126, no. 1 (2026): 149–183, 10.1021/acs.chemrev.5c00454. 41416710

[61] W. Tang , J. P. Fried , R. D. Tilley , and J. J. Gooding , “Understanding and Modelling the Magnitude of the Change in Current of Nanopore Sensors,” Chemical Society Reviews 51, no. 14 (2022): 5757–5776, 10.1039/D1CS00972A. 35748606

[62] Z.‐Q. Zhou , S.‐C. Liu , J. Wang , et al., “Exploring a Solid‐State Nanopore Approach for Single‐Molecule Protein Detection From Single Cells,” Chemical Science 16, no. 19 (2025): 8501–8508, 10.1039/D5SC01764E. 40236591 PMC11996040

[63] P. Fu , B. Chen , Y. Zhang , L. Chen , H. Jeong Lee , and D. Zhang , “Breaking the Diffraction Limit in Molecular Imaging by Structured Illumination Mid‐Infrared Photothermal Microscopy,” Advanced Photonics 7, no. 03 (2025), 10.1117/1.AP.7.3.036003.

[64] S. Wang , P. Fu , H. J. Lee , and D. Zhang , “Laser‐Scanning Infrared‐Raman‐Fluorescence Microscopy for Metabolic Flux Imaging in Living Organisms,” Laser & Photonics Reviews 20, no. 7 (2026): 02715, 10.1002/lpor.202502715.

