# Supplementary material for: Single‐Particle Mid‐Infrared Photothermal Imaging Reveals Hidden Heterogeneity in Real‐World Micro‐ and Nanoplastics
Source: Adv Sci (Weinh). 2026 Apr 27;13(41):e24291. doi: 10.1002/advs.202524291 (PMC13335550; doi:10.1002/advs.202524291)
Supplement: Supplementary file 1 — Supporting File: advs75475‐sup‐0001‐SuppMat.docx. [file ADVS-13-e24291-s001.docx]

Supporting Information

**Single-Particle Mid-Infrared Photothermal Imaging Reveals Hidden Heterogeneity in Real-World Micro- and Nanoplastics**

*Xinyu Deng, Yongqing Zhang, Xiaobin Tang, Hyeon Jeong Lee, Delong Zhang**

This file includes:

Supplementary Note 1. Methods

Supplementary Note 2. COMSOL simulation of transient temperature response of PET particle

Supplementary Note 3. Spectral identification method

Figure S1. Four imaging wavenumbers cover the MIP characteristic peaks of common plastics

Figure S2. The FTIR (black line) and MIP (red line) spectra of 11 common plastics

Figure S3. Image of PMMA microspheres of different sizes to characterize the system

Figure S4. MIP images of particles with different circularity

Figure S5. MIP spectra of PET monomers and standard PET particles

Figure S6. The geometry diagram for the absorption cross section simulation in COMSOL

Figure S7. COMSOL simulation of the temperature evolution of PET particles of different sizes

Figure S8. Assessment of photothermal effects on spectral stability of a single PET particle

Table S1. The wavenumbers of FTIR and MIP characteristic peaks and their corresponding vibration modes of 11 common plastics

Table S2. Peak positions and uncertainties from peak fitting of averaged spectra of PET particles

Table S3. Absorption cross-sections of PET microspheres as a function of particle size, calculated using COMSOL simulations and Mie scattering theory

Table S4. Optimization results comparison of data smoothing for MIP spectra of microplastics

Table S5. Parameters setting in the STV algorithm

Table S6. Identification results of two test spectral sets

Reference

**Supplementary Note 1. Methods**

**1.1. Standard Sample Preparation**

Polymethyl methacrylate (PMMA) microspheres (Wuxi Rigor Biotech) of different sizes (150 nm, 280 nm, 500 nm) were used to evaluate the detection limit of the instrument. 5 μL of each size of microsphere suspension was spin-coated on the surface of calcium fluoride (CaF_2_) substrates for MIP imaging.

Nine standard plastic powder samples with an average particle size of 2 μm, including PMMA, polyvinyl chloride (PVC), polybutylene adipate-co-terephthalate (PBAT), polypropylene (PP), polystyrene (PS), polyethylene (PE), polyethylene terephthalate (PET), polyamide (PA), polylactic acid (PLA), were uniformly deposited on CaF_2_ substrates without further treatment and mounted on the sample stage for spectroscopic analysis. Powdered monomeric species of PET, including bis(2-hydroxyethyl) terephthalate (BHET), terephthalic acid (TPA), and isophthalic acid (IPA), were uniformly deposited on glass slides for spectroscopic detection. Two standard plastic plates, polycarbonate (PC), and polyurethane (PU), were analyzed directly without any pretreatment.

**1.2. Sample Preparation of MNPs in Bottled Water**

Three commercial bottled water brands (PET bottles with PP caps) were obtained from a local supermarket in Hangzhou, China. For each brand, two bottles of water were filtered onto an Al_2_O_3_ membrane filter (Cytiva Whatman, Anodisc 25 mm, 0.02 μm pore-size) using a vacuum filtration apparatus. During the experiments, the researchers wore cotton lab coats and nitrile gloves, and all glassware was rinsed twice with water from the same brand. Throughout the filtration process, the top of the filter funnel was covered with aluminum foil to minimize airborne particle contamination. The foil cover was only removed temporarily when adding water samples. After filtration was completed, each filter membrane was carefully transferred to a glass slide, secured with nail polish around the edges, and placed in a petri dish wrapped aluminum foil to dry overnight before instrumental analysis. During microscopic imaging, an acrylic enclosure (PMMA material) was installed on the sample stage to reduce the contamination from the air. Subsequent analysis confirmed no PMMA particle contamination in the final results. Two blank control groups were set up. One of them was to use Milli-Q water (Nantong Feiyu Biotechnology) instead of bottled water, while all other procedures remained identical to those described above. Another one directly repeats the above steps with a blank filter membrane. A total of three experimental groups and two control groups were established, each subjected to two replicate experiments.

**1.3. MIP Imaging**

MIP images and spectra were acquired using a commercial mIRage microscope (Photothermal Spectroscopy Corp., CA). MIRcat tunable pulsed Quantum Cascade Laser (Daylight Solutions, San Diego, CA) with a pulse frequency of 100 kHz and scanning range of 933–1799 cm^-1^ was used as the pump beam, and a 532 nm continuous-wave laser (Cobolt, 200 mW) was used as the probe beam. The two beams were spatially overlapped using a dichroic mirror and focused onto the sample through a reflective objective (PIKE, 40×/0.78 NA). A motorized scanning stage (MLS203, Thorlabs) was used for sample scanning, and the reflected probe light was collected by the same objective and directed to a photodetector. The signal was demodulated by a lock-in amplifier (Zurich, MFLI) and recorded on a computer.

*1.3.1. Development of a spectral library of standard samples*

We established a standard MIP spectral database of 11 common plastic samples. Spectral scanning was performed at 100 cm^-1^ s^-1^ to obtain high SNR reference spectra, and the spectral resolution was 2 cm^-1^. To determine the laser damage threshold, the IR power was gradually increased from the minimum level at the sample’s most intense absorption peak, while the probe power was adjusted accordingly. The maximum safe power level was established as 20% IR power and 3% probe power with a gain of 5×, which was then used for all measurements of bottled water samples and blank control groups. The four wavenumbers at 1255, 1453, 1471, and 1730 cm^-1^ were selected as they collectively cover the characteristic vibrational peaks of all samples in the spectral library, enabling efficient imaging of real samples in subsequent measurements **(Figure S1)**. In addition, spectroscopic comparisons between MIP and FTIR demonstrate that MIP maintains high spectral fidelity to FTIR while achieving a high SNR **(Figure S2, Table S1)**.

*1.3.2. Determination of System Detection Limit*

The filtration step employed a 20-nm Al_2_O_3_ membrane, which defines the lower bound of particle collection. Particles below this size are not retained and therefore are not represented in subsequent analysis.

Imaging of different-sized PMMA microspheres was performed to determine the system's detection limit **(Figure S3)**. The detection wavenumber was 1730 cm^-1^, the IR power and probe power were set at 71% and 26%, the gain was 5×, the pixel size was 50 nm, and the dwell time was 0.5 ms or 2.5 ms. The system successfully detected 150 nm particles with a signal-to-noise ratio (SNR) of 3.71 in the fingerprint region. We performed Gaussian fitting of the intensity profile from a single 280 nm microsphere which revealed a measurement resolution of 314 nm.

*1.3.3. Detection of bottled water samples*

For each filter membrane of bottled water samples and blank control groups, 13 fields of view with 160 × 120 μm^2^ area were imaged at the four wavenumbers,with a pixel size of 50 nm and pixel dwell time of 0.1 ms. Spectra were scanned at 1000 cm^-1^ s^-1^ and averaged 5 times. The minimal contamination in procedural blanks (Milli-Q water: 2.5 ± 0.4 particles field^-1^, clean Al_2_O_3_ membrane controls: 0.08 ± 0.05 particles field^-1^; *t*-test, *P* < 0.001), which underwent identical sample processing as bottled water samples, validates the reliability of our quantification and demonstrates effective contamination control throughout the analytical workflow.

**1.4. Fourier-Transform Infrared (FTIR) Spectra Acquisition**

The FTIR spectra were acquired in the ATR total reflection mode on a commercial FTIR spectrometer (Nicolet iS10, Thermo Fisher Scientific), with a spectral resolution of 4 cm^-1^ and average 16 times. The spectra were performed advanced ATR correction using Omnic software.

**1.5. Statistical Analysis**

Data processing and statistical analyses were performed using MATLAB R2023a and Origin 2024. Prior to analysis, all spectra were subjected to morphological baseline correction and normalized to the minimum and maximum values unless otherwise stated. For PCA, the data were further mean-centered. No outliers were excluded.

Morphological characteristics of particles were determined in MATLAB using a signal intensity threshold-based method. The threshold for each particle was defined as 1/e of the average of the 20 highest pixel values within the signal region, and the effective area was used to extract morphological parameters. Spectral identification was performed using the spectral total variation (STV) algorithm for noise reduction, followed by μIDENT-based spectral matching (see **Supplementary Note 3** for details).

For particle abundance analysis, the number of particles per field of view was quantified across 2 × 13 fields of view (n = 26). For particle size distribution, measurements were performed on two independent replicates for each brand (n = 2), where each replicate corresponds to a separate experimental measurement. Data are presented as mean ± SEM unless otherwise specified. Statistical significance was assessed using two-sided Student’s *t*-tests with a significance level of α = 0.05.

Curve fitting was performed using Origin with a hybrid Gaussian–Lorentzian (Voigt) cross function model. Initial parameters were determined based on second-derivative spectra and prior knowledge of vibrational modes, and optimized using the Levenberg–Marquardt algorithm. The quality of all fits was rigorously validated, with the adjusted R^2^ values consistently exceeding 0.99, confirming an excellent agreement between the fitted model and the experimental data.

**Supplementary Note 2. COMSOL simulation of transient temperature response of PET particle**

To quantitatively evaluate the transient temperature response of PET microspheres under mid-infrared excitation, finite-element simulations were performed using COMSOL Multiphysics 6.2. The simulation workflow begins by determining the absorption cross section of the particle and subsequently mapping the absorbed energy into a time-dependent thermal response. The simulated mid-infrared wavelength is fixed at 5780.34 nm (1730 cm^-1^). The simulation workflow refers to the steps outlined in the literature [1].

For the absorption cross-section calculation, the geometric model is shown in the **Figure S6**. The microsphere was embedded in air. The outer boundary of the microsphere defines a spherical air region with a radius 20 times that of the particle. The outermost layer has a thickness of 3 times the particle radius, and is a perfectly matched layer designed to suppress reflections from the numerical boundary. The complex refractive index of PET was set to 1.3468 + 0.50516i.^[2]^ The incident field was modeled as a monochromatic plane wave propagating along the z-axis. The absorption cross-section *σ_abs_* was calculated by:

$$\sigma_{abs}=\frac{1}{I}\iiint Q_{e}dV$$

where *Q_e_* (ewfd.Q_e_ in COMSOL) is the electromagnetic power loss density and *I* is the incident intensity. To validate the numerical accuracy, the simulated absorption cross-sections were benchmarked against analytical results derived from Mie scattering theory. Excellent agreement between the two confirms the reliability of the numerical discretization **(Table S3)**.

For the photothermal dynamics simulation, the absorbed optical power was converted into a heat source term to drive the thermal simulation. The heat source is given by:

$$Q\left( t \right)=\frac{P{\cdot\sigma}_{abs}}{V}pulse(t)$$

where *P* is the laser power density, set to 2.7E7 W m^-2^, *V* is the volume of the microsphere and *pulse(t)* represents the normalized temporal profile of the excitation pulse, which was defined as a pulsed Gaussian waveform with a pulse duration of 500 ns. Because the particle size is much smaller than the excitation wavelength, the heat source can be reasonably approximated as spatially homogeneous within the particle. The temperature evolution of the system was governed by the time-dependent heat conduction equation:

$$\rho C_{p}\frac{\partial T}{\partial t}-\nabla\cdot\left( k\nabla T \right)=Q\left( t \right)$$

where *ρ*, *C_p_*, and *k* denote the density, heat capacity, and thermal conductivity of the materials, respectively. The simulation domain included the PET microsphere, the surrounding air, and the glass substrate. To account for realistic thermal contact, a contact region with a radius of 0.4 times the particle radius was introduced between the particle and the substrate. The initial temperature of the system was set to 298 K. The transient temperature response was obtained by solving the heat equation over time, and the maximum temperature of the microsphere was extracted **(Figure S7)**. The results show that the maximum temperature rise remains on the order of 8 K.

**Supplementary Note 3: Spectral identification method**

3.1. Spectral Total Variation algorithm

STV algorithm is a supervised denoising method designed for hyperspectral images^[3]^. It formulates the denoising task as a numerical optimization problem with the following objective function:

$$\min_{\mathbf{f}} \frac{1}{2}\parallel\mathbf{f}-\mathbf{g}\parallel^{2}+\parallel\mathbf{w}\odot\left( \mathbf{Df} \right)\parallel_{1}, \mathbf{D}=\left[ \begin{aligned} \beta_{x}\mathbf{D}_{x} \\ \beta_{y}\mathbf{D}_{y} \\ \beta_{\lambda}\mathbf{D}_{\lambda} \end{aligned} \right].$$

Here, **g** represents the vector of the observed (noisy) image, and **f** denotes the vector of the desired (clean) image. The objective function comprises two terms: (i) a quadratic term that quantifies the residual between the observed image and the solution, ensuring data fidelity, and (ii) a total variation term applied to the solution, which promotes spatial smoothness. The influence of the spatial and spectral derivatives, **D***_x_*, **D***_y_*, and **D***_λ_*, is modulated by the parameters *β_x_*, *β_y_*, and *β_λ_*. When both *β_x_* and *β_y_* are set to zero while *β_λ_* remains non-zero, the formulation corresponds to spectral smoothing along the wavelength dimension. The parameter **w** serves as a weighting factor that balances the relative contribution of each term.

Since noise levels typically vary across spectral bands, distinct values of **w** are assigned to each band. The values of **w** can be determined based on an estimation of the noise level:

$$\mathbf{w}=\left( \sigma_{1}^{\alpha},\sigma_{2}^{\alpha},\ldots,\sigma_{k}^{\alpha} \right), \sigma_{k}=\left( \sum_{i} \left( f_{k,i}-\bar{f_{k}} \right)^{2} \right)^{1/2},$$

where power constant *α* satisfies 1* *≤* α ≤* , and *σ*_1_*^α^** *_2_*^α^* … * σ_k_^α^* correspond to the weights for different spectral bands. Here, $\bar{f_{k}}$ denoted the mean value of the noisy spectral band *k*. Finally, the above optimization problem is solved using the Alternating Direction Method of Multipliers (ADMM).

3.2 Optimization of Data Preprocessing Algorithms

Prior to spectral identification, data smoothing was applied to the raw spectra. For the spectral data acquired by the MIP system, the optimal smoothing algorithm and its corresponding parameters were selected. A total of 352 test spectra were constructed from 11 reference spectra and 8 noise spectra, with SNR of 10, 20, 30, and 40 dB, to optimize the parameters of each algorithm. The objective function was defined as minimizing the normalized root mean square error (nRMSE) between the smoothed spectra and the reference spectra. All input parameters for the algorithms were discretized, and a grid search was employed to identify the optimal parameter values. The output metric was the average nRMSE achieved by each algorithm. The tested algorithms, along with their best target values, are summarized in the **Table S4**. The results indicate that the STV algorithm achieves the smallest nRMSE among all compared methods, demonstrating the best overall performance. Therefore, the STV algorithm will be employed for spectral data preprocessing in subsequent analyses, with specific parameter configurations provided in **Table S5** (unspecified parameters were set to their default values; please refer to [3] for a detailed description).

3.3. Spectral Identification

Microplastics identification algorithm (μIDENT) was employed for spectral matching^[4]^, wherein the original spectral preprocessing step was replaced with the STV algorithm. The matching performance of the algorithm was evaluated using two sets of test spectra in the context of MIP-based spectral matching. The first test set consisted of 352 spectra, which were also used in the data preprocessing optimization step. These spectra were matched against 11 reference spectra. The second test set comprised 122 spectra collected from bottled water, each manually annotated with ground truth labels. Most of these spectra were identified as PET, with a minority being recognized as PP. It is worth noting that due to excessively strong noise around the 1200 cm^-1^ region, the spectral segment between 1200–1244 cm^-1^ was excluded during matching. Furthermore, as discussed in the main text, PET particles extracted from bottled water exhibited significant aging effects, manifesting as peak splitting across multiple spectral bands. To account for this, a high SNR spectrum of aged PET was included in the reference set, bringing the total number of reference spectra to 12 for the second test set. The empirical parameters in the matching process, denoted as k_1_, k_2_, k_3_, and k_4_, were set to 1.2, 8.4, 3.0, and 4.7, respectively, and consistently applied across all matching process. The performance was quantitatively assessed in terms of *correctly–identified–rate* (CIR), *not–identified–rate* (NIR), and *incorrectly–identified–rate* (IIR). As summarized in **Table S6**, both test sets achieved correct identification rates exceeding 95%. Nevertheless, given the possibility of misidentification, the μIDENT matching results for real-world samples will be subject to manual verification.

**Figure S1.** **Four imaging wavenumbers cover the MIP characteristic peaks of common plastics.** The vertical gray lines from left to right correspond to 1255, 1453, 1471, 1730 cm^-1^, which represent the characteristic infrared peaks of the 11 common plastics.

**Figure S2.** **The FTIR (black line) and MIP (red line) spectra of 11 common plastics.** (a) polymethyl methacrylate (PMMA), (b) polypropylene (PP), (c) polybutylene adipate-co-terephthalate (PBAT), (d) polycarbonate (PC), (e) polylactic acid (PLA), (f) polyethylene (PE), (g) polyethylene terephthalate (PET), (h) polyurethane (PU), (i) polyvinyl chloride (PVC), (j) polystyrene (PS), (k) polyamide (PA). Among these, the samples in (a)-(c), (e)-(g), (i)-(k) are in powder form, and the samples in (d) and (h) are in block form.

**Figure S3.** **Image of PMMA microspheres of different sizes to characterize the system.** (a)-(c) The MIP images of 500 nm, 280 nm and 150 nm PMMA microspheres at 1730 cm^-1^. Scale bar: 2 μm. (d)-(f) Corresponding DC images of (a)-(c). (g) The single 280 nm PMMA within the yellow dashed box in (b). Scale bar: 0.5 μm. (h) Resolution of the system obtained by Gaussian fitting (red line) of the profile (black dots) corresponding to the yellow dashed line in (g).

**Figure S4. MIP images of particles with different circularity.** (a)-(d) display particle images with progressively increasing circularity. It can be observed that particles with lower circularity appear more elongated and irregular in shape. Conversely, as the circularity value approaches 1, the particle shape becomes increasingly circular.

**Figure S5. MIP spectra of PET monomers and standard PET particles.** BHET: bis(2-hydroxyethyl) terephthalate, TPA: terephthalic acid, IPA: isophthalic acid. Solid lines represent the mean of the normalized spectra for each substance, and shaded areas indicate the interquartile range. The vertical grey line marks the reference position at 1690 cm^-1^.

**
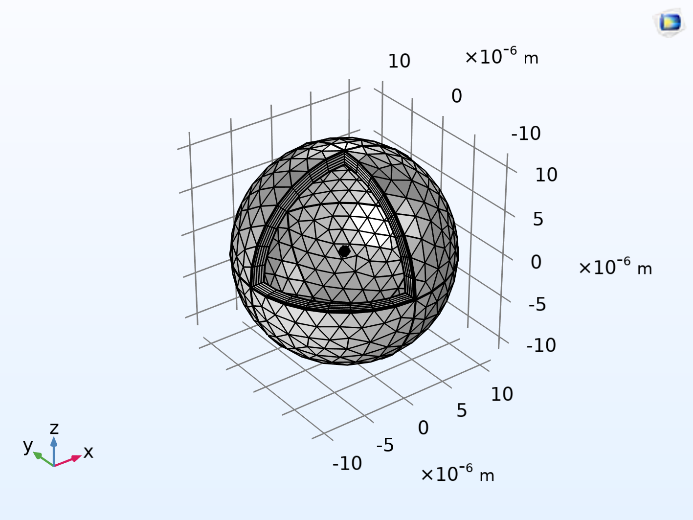
**

**Figure S6. The geometry diagram for the absorption cross section simulation in COMSOL.** The central part is the PET particle. The surrounding region is air with a radius 20 times that of the particle. The outermost layer is the perfectly matched layer with a thickness of 3 times the particles radius.

**Figure S7. COMSOL simulation of the temperature evolution of PET particles of different sizes.**

**Figure S8. Assessment of photothermal effects on spectral stability of a single PET particle.** (a) Sequential MIP spectra acquired at a fixed location over 220 s (22 spectra in total). (b) Pearson correlation coefficients of the 21 subsequent spectra as compared with the first spectrum.

**Table S1. The wavenumbers of FTIR and MIP characteristic peaks and their corresponding vibration modes of 11 common plastics.**

|  | FTIR (cm^-1^) | MIP (cm^-1^) | Assignment^[5–11]^ |
| --- | --- | --- | --- |
| **PMMA** | 1149 | 1152 | C-O-C symmetric stretching |
|  | 1193 | 1195 | C-O-C symmetric stretching |
|  | 1242 | 1244 | C-O-C antisymmetric stretching |
|  | 1269 | 1271 | C-O-C antisymmetric stretching |
|  | 1436 | 1437 | C-H bending vibration |
|  | 1481 | 1483 | C-H bending vibration |
|  | 1729 | 1734 | C=O stretching |
| **PP** | 973 | 973 | C-C bond vibration |
|  | 999 | 999 | C-H bond vibration |
|  | 1168 | 1168 | C-H bond vibration |
|  | 1376 | 1377 | CH_3_ bending vibration |
|  | 1457 | 1457 | CH_3_ bending vibration |
| **PBAT** | 1019 | 1019 | =C-H in-plane bending in benzene ring |
|  | 1106 | 1106 | C–O stretching |
|  | 1124 | 1122 | C–O stretching in alphabetic acid |
|  | 1172 | 1167 | C–O stretching in alphabetic acid |
|  | 1276 | 1274 | C–O stretching |
|  | 1410 | 1410 | trans-CH2-plane bending vibration |
|  | 1504 | 1506 | skeleton vibration of the benzene ring |
|  | 1723 | 1723 | C=O stretching vibration |
| **PC** | 1015 | 1015 | C-O-C stretching |
|  | 1082 | 1081 | C-O-C stretching |
|  | 1164 | 1164 | C-O-C stretching |
|  | 1194 | 1195 | C-O-C stretching |
|  | 1231 | 1231 | C-O-C stretching |
|  | 1505 | 1505 | aromatic ring vibration |
|  | 1774 | 1777 | C=O stretching |
| **PLA** | 1102 | 1102 | C-O bond vibration |
|  | 1134 | 1134 | C-O bond vibration |
|  | 1184 | 1186 | C-O bond vibration |
|  | 1380 | 1381 | methyl vibration |
|  | 1725 | 1728 | C=O stretching |
| **PE** | 1471 | 1471 | CH_2_ deformation vibration |
| **PET** | 1019 | 1021 | C-H in-plane stretching |
|  | 1099 | 1103 | C-O-C stretching |
|  | 1121 | 1124 | C-O-C stretching |
|  | 1249 | 1257 | ester group stretching |
|  | 1341 | 1341 | CH_2_ wagging vibration |
|  | 1409 | 1410 | C-H aromatic ring vibration |
|  | 1716 | 1722 | C=O stretching |
| **PU** | 1530 | 1530 | N-H |
|  | 1601 | 1601 | C=C |
|  | 1726 | 1731 | C=O stretching |
| **PVC** | 1089 | 1105 | C-C stretching |
|  | 1252 | 1255 | C-H bending |
|  | 1426 | 1427 | C-H aliphatic bending |
| **PS** | 1029 | 1029 | aromatic ring in-plane CH bending |
|  | 1452 | 1453 | CH_2_ deformation vibration |
|  | 1492 | 1493 | vibrational mode of aromatic rings |
|  | 1601 | 1602 | vibrational mode of aromatic rings |
| **PA** | 1467 | 1466 | N-H and C-N amide II stretching |
|  | 1552 | 1566 | N-H and C-N amide II stretching |
|  | 1640 | 1642 | C=O amide I stretching |

**Table S2. Peak positions and uncertainties from peak fitting of averaged spectra of PET particles.**

| Assignment | Standard PET [cm^-1^] | PET particle [cm^-1^] |
| --- | --- | --- |
| C=O stretching | 1734.0 ± 0.9 | 1730.2 ± 0.1 |
|  | 1722.8 ± 0.3 | 1718.5 ± 0.1 |
|  | 1688.0 ± 2.1 | - |
| Ester group stretching | 1290.7 ± 0.1 | 1288.2 ± 0.7 |
|  | 1267.8 ± 0.1 | 1263.6 ± 0.4 |
|  | 1252.5 ± 0.2 | 1246.5 ± 0.2 |

**Table S4.** **Optimization results comparison of data smoothing for MIP spectra of microplastics.**

| Data Smoothing Algorithm | nRMSE (%) |
| --- | --- |
| Moving Average | 2.16 |
| Discrete Wavelet Transform | 3.03 |
| Savitzky-Golay filter | 2.19 |
| Savitzky-Golay filter (variable smoothing span) | 2.18 |
| Spectral Total Variation | 1.67 |

**Table S5. Parameters setting in the STV algorithm.**

| Parameter | Description | values |
| --- | --- | --- |
| opts.tv_method | Total variation method | ‘aniso’ |
| opts.rho_r | Initial penalty parameter for \|\| u−Df \|\| | 1 |
| opts.rho_o | Initial penalty parameter for \|\| f−g−r \|\| | 20 |
| opts.beta | [*β_x_*_,_ *β*__, *β*_λ_] | [0 0 1.2] |
| opts.tol | Tolerance level on relative change | 5e-2 |
| α | power constant α | 0.7 |

**Table S6. Identification results of two test spectral sets.**

|  | CIR | NIR | IIR |
| --- | --- | --- | --- |
| Case 1 (n=352) | 96.31 | 1.70 | 1.99 |
| Case 2 (n=122) | 95.08 | 4.92 | 0.00 |

Reference

1. Zong, H., C. Yurdakul, Y. Bai, et al., "Background-Suppressed High-Throughput Mid-Infrared Photothermal Microscopy via Pupil Engineering," *ACS Photonics* *8*, no.11 (2021): 3323. https://doi.org/10.1021/acsphotonics.1c01197

2. Zhang, X., J. Qiu, J. Zhao, X. Li, L. Liu, "Complex Refractive Indices Measurements of Polymers in Infrared Bands," *Journal of Quantitative Spectroscopy and Radiative Transfer* *252*, (2020): 107063. https://doi.org/10.1016/j.jqsrt.2020.107063

3. Liao, C.-S., J. H. Choi, D. Zhang, S. H. Chan, J.-X. Cheng, "Denoising Stimulated Raman Spectroscopic Images by Total Variation Minimization," *The Journal of Physical Chemistry C* *119*, no.33 (2015): 19397. https://doi.org/10.1021/acs.jpcc.5b06980

4. Renner, G., P. Sauerbier, T. C. Schmidt, J. Schram, "Robust Automatic Identification of Microplastics in Environmental Samples Using FTIR Microscopy," *Analytical Chemistry* *91*, no.15 (2019): 9656. https://doi.org/10.1021/acs.analchem.9b01095

5. Böke, J. S., J. Popp, C. Krafft, "Optical Photothermal Infrared Spectroscopy with Simultaneously Acquired Raman Spectroscopy for Two-Dimensional Microplastic Identification," *Scientific Reports* *12*, no.1 (2022): 18785. https://doi.org/10.1038/s41598-022-23318-2

6. Tarafdar, A., J. Xie, A. Gowen, A. C. O’Higgins, J.-L. Xu, "Advanced Optical Photothermal Infrared Spectroscopy for Comprehensive Characterization of Microplastics from Intravenous Fluid Delivery Systems," *Science of The Total Environment* *929*, (2024): 172648. https://doi.org/10.1016/j.scitotenv.2024.172648

7. Cai, Y., J. Lv, J. Feng, "Spectral Characterization of Four Kinds of Biodegradable Plastics: Poly (Lactic Acid), Poly (Butylenes Adipate-Co-Terephthalate), Poly (Hydroxybutyrate-Co-Hydroxyvalerate) and Poly (Butylenes Succinate) with FTIR and Raman Spectroscopy," *Journal of Polymers and the Environment* *21*, no.1 (2013): 108. https://doi.org/10.1007/s10924-012-0534-2

8. Chen, Z., J. N. Hay, M. J. Jenkins, "FTIR Spectroscopic Analysis of Poly(Ethylene Terephthalate) on Crystallization," *European Polymer Journal* *48*, no.9 (2012): 1586. https://doi.org/10.1016/j.eurpolymj.2012.06.006

9. Pandey, M., G. M. Joshi, A. Mukherjee, P. Thomas, "Electrical Properties and Thermal Degradation of Poly(Vinyl Chloride)/Polyvinylidene Fluoride/ ZnO Polymer Nanocomposites," *Polymer International* *65*, no.9 (2016): 1098. https://doi.org/10.1002/pi.5161

10. Kherroub, D., M. Belbachir, S. Lamouri, L. Bouhadjar, K. Karim Chikh, "Synthesis of Polyamide-6/Montmorillonite Nanocomposites by Direct In-Situ Polymerization Catalysed by Exchanged Clay," *Oriental Journal of Chemistry* *29*, no.4 (2013): 1429. https://doi.org/10.13005/ojc/290419

11. Reghunadhan, A., S. Thomas, "Polyurethanes,". In *Polyurethane Polymers*; Elsevier. Press, 2017; pp 1–16
